# Sex disparities in dialysis initiation, access to waitlist, transplantation and transplant outcome in German patients with renal disease—A population based analysis

**DOI:** 10.1371/journal.pone.0241556

**Published:** 2020-11-12

**Authors:** Anette Melk, Bernhard M. W. Schmidt, Siegfried Geyer, Jelena Epping

**Affiliations:** 1 Department of Pediatric Kidney, Liver and Metabolic Diseases, Hannover Medical School, Hannover, Germany; 2 Department of Nephrology and Hypertension, Hannover Medical School, Hannover, Germany; 3 Department of Medical Sociology, Hannover Medical School, Hannover, Germany; Imperial College Healthcare NHS Trust, UNITED KINGDOM

## Abstract

**Background:**

Renal transplantation access and outcome differ between men and women, but no analysis has considered all transition phases and transplant outcome using the same data set. We analyzed sex disparities in all phases of patients’ clinical path (progression to dialysis, waitlisting, transplantation, graft failure/death).

**Methods:**

In a population based approach using health insurance data (2005–2013) we examined patients’ risk of changing from one phase to another applying Cox Proportional Hazards model.

**Results:**

After adjusting for age and comorbidities, women had a 16% lower risk of progression to ESRD (HR/95%-CI: 0.84/0.79–0.88). Access to the waitlist was lowered by 18% in women compared to men (HR/95%-CI: 0.82/0.70–0.96). An age stratified analysis did not reveal differences in any age group. Once waitlisted, the chance to receive a transplant was identical (HR/95%-CI: 0.96/0.81–1.15). The risk of transplant failure/death was identical for both sexes (HR/95%-CI: 0.99/0.73–1.35), but the effect was modified by age: in younger women (18–45 years) the risk was twice as high compared to men (HR/95%-CI: 2.08/1.04–4.14), whereas the risk in elderly women (> 65 years) was only half the risk of men (HR/95%-CI: 0.47/0.24–0.93).

**Conclusion:**

Sex disparities occurred at different steps in the history of patients with renal disease and affected progression to dialysis, waitlisting and transplantation outcome in a population with equal access to medical treatment.

## Introduction

Kidney transplantation is the treatment of choice in patients reaching end stage renal disease (ESRD). There are conflicting results whether access to transplantation differs between male and female patients. Some studies have shown that the likelihood of women undergoing kidney transplantation is lower than that of men [1–3], whereas others have shown no sex disparity [4, 5]. The latter studies have used overlapping samples from the same data source of UK Renal Registry, while the other studies were conducted in the US. The data on outcome after transplantation is mainly derived from registry data and tend to show that male recipients have a better outcome [6–8].

Kidney transplantation is the endpoint of a process that starts with declining renal function. Patients with chronic kidney disease (CKD) reaching ESRD undergo several transition phases, if they do not receive a pre-emptive transplantation: initiation of dialysis, placement on the transplant waiting list, renal transplantation and eventually graft failure or death. With each transition to the next step the number of individuals gets smaller with only a fraction of patients finally receiving a transplant. Most of the available studies consider only one or two of the several transition phases that lead to transplantation, but none has followed patients all the way to report on outcome. It remains to be shown whether sex disparities occur at different steps in this sequence and if they affect transplantation outcome.

The incidence of the first transition, i.e. starting renal replacement therapy (RRT, either dialysis or transplantation), is mainly determined by progression of renal disease. There is experimental and epidemiological evidence that progression of renal diseases is faster in men than in women [9]. Besides age, baseline glomerular filtration rate (GFR) and urinary albumin excretion, male sex is an important factor that determines progression of renal disease [10]. It is assumed that renoprotective effects of female sex hormones are causative for this difference in disease progression [11].

Since the late 1980s it was almost uniformly reported that women have reduced access to renal transplantation [1–5, 12–19]. While several studies only investigated access to the transplant waiting list [3, 4, 13–16] only a few studies addressed access to transplantation after waitlisting [2, 5, 17, 18], but with inconsistent results. From studies performed within the United States (US) health care system it had been concluded that socio-economic factors are the underlying cause [20, 21], because the lower average income of women is associated with inferior health care plans and lower quality of care in the US. This economic explanation of sex differences, however, was challenged by European studies, e.g. from France and Scotland, where universal health care coverage exists, but similar sex differences were reported for access to the waiting list [16, 18]. This might be because financial coverage does not omit other components of socio-economic status like self-efficacy, patient activation, health literacy, social support and clinician bias [18].

Studies on outcome after renal transplantation based on the recipient’s sex are much more difficult to interpret because there are conflicting data on the potential influence of the donor’s sex [6–8, 22–27]. In general, most studies have suggested that patient and graft survival is superior in men when compared to women [6–8], whereas few studies have shown a better 10 year-mortality rate in female recipients or no difference in death censored graft survival [25, 26], respectively. Recently, an elegant study showed that graft survival is only superior in women over men in female recipients over 45 years of age transplanted with a female donor kidney pointing towards the importance of including recipient age in such analyses [27].

Because none of the previous studies had described all transition phases as well as transplantation outcome, our recent study takes a population based approach to address this problem and to overcome the limitations of registry studies. Using data from a large German statutory health insurance we investigated sex disparities for each important phase in a patient’s clinical path (initiation of dialysis, placement on the transplant waiting list, renal transplantation, graft failure/death) considering age and existing comorbidities as important cofounders. With Germany being a country providing full health care coverage to everybody without financial barriers to medical care, we ascertain to minimize the influence of socio-economic factors on the reported results. In addition, systematic data focussing on access to or result of renal transplantation from Germany have not been available yet [28].

### Patients and methods

Our analyses are based on pseudonymised health insurance data of a local statutory health insurance, the AOK (*Allgemeine Ortskrankenkasse*) Lower Saxony, covering the years 2005 to 2013 with a total study population of 3 million women and men aged 18 years and older from the state of Lower Saxony of whom patients were identified as suffering from renal disease as defined below. In Germany health insurance coverage is obligate for all residents with only less than 0.2% of residents being without coverage [29]. Below a certain income level, coverage through statutory health insurance is obligate. Health care coverage is the same for all insured individuals within the statutory health care system and regular adjustments are made to account for new developments and medical progress. With regard to dialysis and transplantation, all costs are completely covered. Privately insured individuals are not included in our data. Private health insurances are only covering state- and self-employed individuals and those in the upper 10% of the income distribution making up 14% of employed residents [30]. The socio-demographic structure of the individuals insured through AOK closely resembles that of whole Germany, but individuals of lower income groups are over-represented [31].

The AOK data contain information on in- and outpatient treatments (according to payment codes, so called “Gebührenordnungspositionen” and according to treatment codes, so called *Operationen und Prozeduren Schlüssel*, OPS codes) with diagnoses (according to the International Classification of Diseases, Tenth Revision, ICD-10), medications, and socio-demographic variables. For each patient we were able to retrieve data collected by the insurance in 3-monthly intervals. As we used only anonymized claims data in our study ethical approval was not necessary.

**Fig 1:**
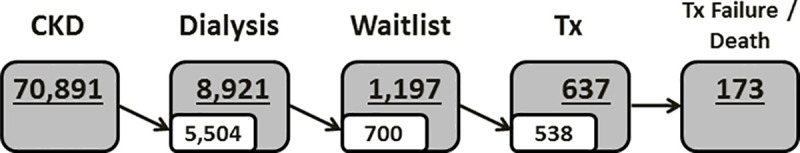
Number of patients in the respective subpopulations used for the analysis.

We identified four subpopulations in order to investigate the proposed transition phases and outcome: (1) CKD, (2) dialysis, (3) waitlist, (4) transplantation (Fig 1). These subpopulations were defined according to the following criteria. CKD patients were identified by outpatient ICD-10 codes N18.1 to N18.5. Patients were defined as being on chronic dialysis treatments if at least one of three relevant payment codes (13611, 13610 or 13602) was assigned. Patients on the waitlist were identified by repeated testing for panel reactive antibody (PRA; payment code: 32530), as for patients on the renal transplant waiting list it is mandatory to undergo PRA testing every 3 months. Subjects who underwent kidney transplantation were identified by OPS code 5–555. Only very few patients (N = 27) were preemptively transplanted (i.e. transplantation without prior dialysis), which reflects clinical reality in adult patients in Germany. These patients only contributed to the last step of the analyses (see below). For the populations “CKD”, “dialysis” and “waitlist”, it was mandatory that the respective codes were present in two sequential 3-months periods. As these codes are mandatory for reimbursement of physicians’, hospitals’ and laboratories’ services, the information on dialysis, transplantation and PRA measurement are very accurate.

The available numbers in the starting group for each transition step are shown in large grey boxes. The smaller white boxes indicate the incident cases that fulfilled the definition for transition into the next phase.

The data are right and left censored. The observation time started on January 1^st^, 2005 and ended on December 31^st^, 2013. Insured persons entering the AOK later than January 1^st^, 2005 were left-censored. Those individuals leaving the AOK before the end of the observation period (lost to follow up) were right-censored. There were no other censoring events. For other individuals the observation time ended with the observed event or at the right-censoring date of December 31^st^, 2013. If patients rejoined the insurance, data of the second period were not used for analysis to avoid interval-censoring.

For our Cox regression analyses, we used the following starting and endpoints. 1.) Transition from CKD to dialysis: Starting point was the first occurrence of a CKD code (N18.1–5) in two sequential 3-months periods of a year and the endpoint of this line of analysis was the occurrence of the payment codes indicating dialysis treatment (13611, 13610 or 13602) in two sequential 3-months periods of a year. 2.) Transition from dialysis to waitlisting: Starting point was the occurrence of dialysis payment codes (13611, 13610 or 13602) and the endpoint was the first occurrence of the payment code for PRA detection (32530), if it was billed in two sequential 3-months periods of a year. Patients were assumed to be waitlisted if in need of regular measurements of PRA to assure their waitlisting status. 3.) Transition from waitlisting to transplantation: Starting point was waitlisting, i.e. PRA detection (32530) and the endpoint of this line of analysis was the occurrence of the OPS code for renal transplantation (5–555). 4.) The OPS code for renal transplantation (5–555) served then as starting point for the final analysis of transplant outcome. Endpoints for this outcome analysis were defined as restarting of dialysis treatment (OPS codes 13611, 13610, 13602) or death. Transplanted patients without the event of dialysis treatment or without death were right censored at the end of the observation period.

Comorbidities were identified by the respective codes used during outpatient care with diabetes mellitus identified through ICD-10 codes E10 to E14, ischemic heart disease through I20 to I25, and cerebrovascular disease through I60 to I69.

We performed a post-hoc power calculation looking at the power of our analyses to detect the actual HR or a decrease or increase of hazard by 10% and 20%, respectively (S1 Table).

### Statistical procedures

Four separate lines of analysis were performed: transitions 1) from CKD to dialysis, 2) from dialysis to waitlist, 3) from waitlist to transplantation and 4) from transplantation to transplantation failure or death (Fig 1). The number of cases at the starting point of each line was defined anew. This means that e.g. for the second analysis step “dialysis to waitlist” not only the incident dialysis cases were used, which built the outcome group in the first analysis step, but all available (prevalent) cases on dialysis (a total of 8,921 individuals). The same applied for analyzing “waitlist to transplantation” and “transplantation to transplant failure/death”.

The risk for proceeding from one state into the next state was examined by means of regression analyses using Cox Proportional Hazards model [32]. Effects are presented in terms of hazard ratios with 95% confidence intervals. The proportional hazards assumption was examined by plotting Kaplan-Meier curves. Data were left and right censored, as patients can join and leave the insurance at any time. If patients left the insurance and entered later again, they participated only with his/her first period. This occurred in only 408 cases. Two-Sample Z-test for proportions was used for the comparison of prevalences of comorbidities in the different groups.

The predefined covariates used for adjusting the sex effect were age as well as the comorbidities diabetes mellitus (ICD-10 E10 to E1), ischemic heart disease (IHD, ICD-10 I20 to I25) and cerebrovascular disease (CVD, ICD-10 I60 to I69). These covariates were used for adjusting the sex effect in each line of analysis. Stratification for age groups (18–45 years, >45–65 years and >65 years) was used to explore effects over different ages. In addition, formal testing for an interaction (sex*age) was performed.

All statistical analyses were performed using STATA 11MP (StataCorp LLC, College Station, USA).

## Results

Fig 1 and Table 1 depict our study population and the demographics of patients identified for each subpopulation. The proportion of women differs for the subpopulations and is lowest for the patients identified as being listed for renal transplantation (36.7% in the waitlisted patients vs. 50.7% in the CKD and 44.1% in the dialysis population; p<0.001, X^2^ test). Mean age is higher for female patients with CKD (74.1 ± 13 vs 69.8 ± 13, p<0.001, t-test) or on dialysis (69.6 ± 14 vs. 66.2 ± 14.2, p<0.001, t-test), but not for the populations on waitlist (48.8 ± 12.6 vs. 49.8 ± 13, p = 0.19, t-test) and after transplantation (50.3 ± 13.2 vs. 50.9 ± 13.5, p = 0.58, t-test).

**Table 1:** Study population.

	CKD		Dialysis		Waitlist		Transplantation	
**N**	70,891		8,921		1,197		637	
**% women**	50.7%		44.1%		36.7%		38.0%	
**Age (M±SD)**								
All	72.0 ± 13.2		67.7 ± 14.2		49.4 ± 12,8		50.6 ± 13.4	
Men	69.8 ± 13.0		66.2 ± 14.2		49.8 ± 13.0		50.9 ± 13.5	
Women	74.1 ± 13.0		69.6 ± 14.0		48.8 ± 12.6		50.3 ± 13.2	
**% with diabetes**	46.3%		50.9%		22.8%		21.7%	
**% with IHD**	40.6%		41.9%		18.7%		20.4%	
**% with CVD**	21.6%		20.5%		7.9%		10.7%	
**% with diabetes, m/f**	46%	47%	49%	53%	25%	20%	23%	20%
**% with IHD, m/f**	45%	37%	44%	39%	21%	14%	23%	17%
**% with CVD, m/f**	22%	21%	22%	19%	9%	7%	11%	10%

IHD, ischemic heart disease; CVD, cerebrovascular disease.

With the exception of diabetes mellitus, men with CKD (IHD: 45% vs 37%, p<0.001, X^2^ test, CVD 22% vs 21%, p = 0.0012, X^2^ test,) or on dialysis (IHD: 44% vs 39%, p<0.001, X^2^ test, CVD 22% vs 19%, p<0.001, X^2^ test,) showed higher comorbidity rates than women. Similar differences can be seen for patients on the waitlist (IHD: 21% vs 14%, p<0.001, X^2^ test, CVD 9% vs 7%, p = 0.25, X^2^ test,) or after transplantation (IHD: 23% vs 17%, p = 0.065, X^2^ test, CVD 11% vs 10%, p = 0.699, X^2^ test,), but at a much lower level. Patients who accessed the waitlist or received transplantation displayed fewer comorbidities when compared to CKD patients and patients on dialysis. E.g. ischemic heart disease (IHD) was prevalent in 20.4% of transplanted patients but in 40.6% of patients with CKD and 41.9% of patients on dialysis (p<0.001, X^2^ test). There is a greater decrease in the comorbidity burden for women with the waitlisting step. While in males the proportion of diabetic patients decreased by 49% (from 49% to 25%), the proportion decreased by 62% in females (from 53% to 20%). The same tendency is seen for ischemic heart disease with the proportion decreasing by 52% in males (44% to 21%) and 64% (39% to 14%) in females.

### CKD to dialysis

For the progression from *CKD to dialysis* (Table 2) a marked sex effect emerged as the risk of women receiving this therapy was 27% lower than that of men (hazard ratio (HR)/95%-confidence interval (CI): 0.73/0.69–0.77). While correction of the model by age led to a slight reduction of disparity due to sex (HR/95%-CI: 0.82/0.78–0.86), the introduction of the investigated comorbidities did not result in a further change of the HR). The likelihood of women starting dialysis remained 16% lower than of men in the adjusted model (HR/95%-CI: 0.84/0.79–0.88). Irrespective of the sex difference, the presence of comorbidities increased the likelihood of progression to dialysis.

**Table 2:** Proportional hazards regression models for the four transition phases.

	CKD^1^ -> Dialysis			Dialysis -> Waitlist			Waitlist -> Tx			Tx ->Failure		
Case numbers	70,793 -> 5,504			8,921 -> 700			1,197 -> 538			637 -> 173		
	**Model 1**	**Model 2**	**Model 3**	**Model 1**	**Model 2**	**Model 3**	**Model 1**	**Model 2**	**Model 3**	**Model 1**	**Model 2**	**Model 3**
**Sex (female vs. male)**	0.73***	0.82***	0.84***	0.65***	0.84*	0.82*	0.96	0.95	0.96	0.99	0.99	0.99
95% CI	0.69–0.77	0.78–0.86	0.79–0.88	0.56–0.76	0.72–0.98	0.70–0.96	0.81–1.14	0.80–1.13	0.81–1.15	0.72–1.34	0.73–1.35	0.73–1.35
**Age (10 years)**		0. 73***	0. 75***		0.27**	0.45**		0.88**	0.85**		1.20**	1.13*
95% CI		0.72–0.75	0.73–0.81		0.23–0.32	0.41–0.50		0.82–0.95	0.79–0.92		1.07–1.32	1.002–1.27
**Diabetes**			1.67***			0.72**			1.08			0.92
95% CI			1.58–1.76			0.60–0.87			0.87–1.34			0.63–1.33
**IHD**			1.44***			0.98			1.04			1.47*
95% CI			1.36–1.53			0.79–1.20			0.82–1.30			1.02–2.13
**CVD**			1.30***			0.90			1.42*			1.57*
95% CI			1.22–1.39			0.69–1.18			1.07–1.88			1.07–1.88

Hazard ratios and 95% confidence intervals from regression analyses using Cox Proportional Hazards model are displayed. Model 1 only includes sex, model 2 includes sex and age and model 3 includes sex, age and the comorbidities diabetes, IHD and CVD. Significant results are marked

* p<0.05 and

** p<0.001. IHD, ischemic heart disease; CVD, cerebrovascular disease; Tx, transplantation. ^1^Restriction of the analysis to patients with CKD stages 4 and 5 (ICD-10: N18.4 or N18.5; N = 21,856) shows essentially the same results (see S2 Table).

### Dialysis to waitlist

**Fig 2:**
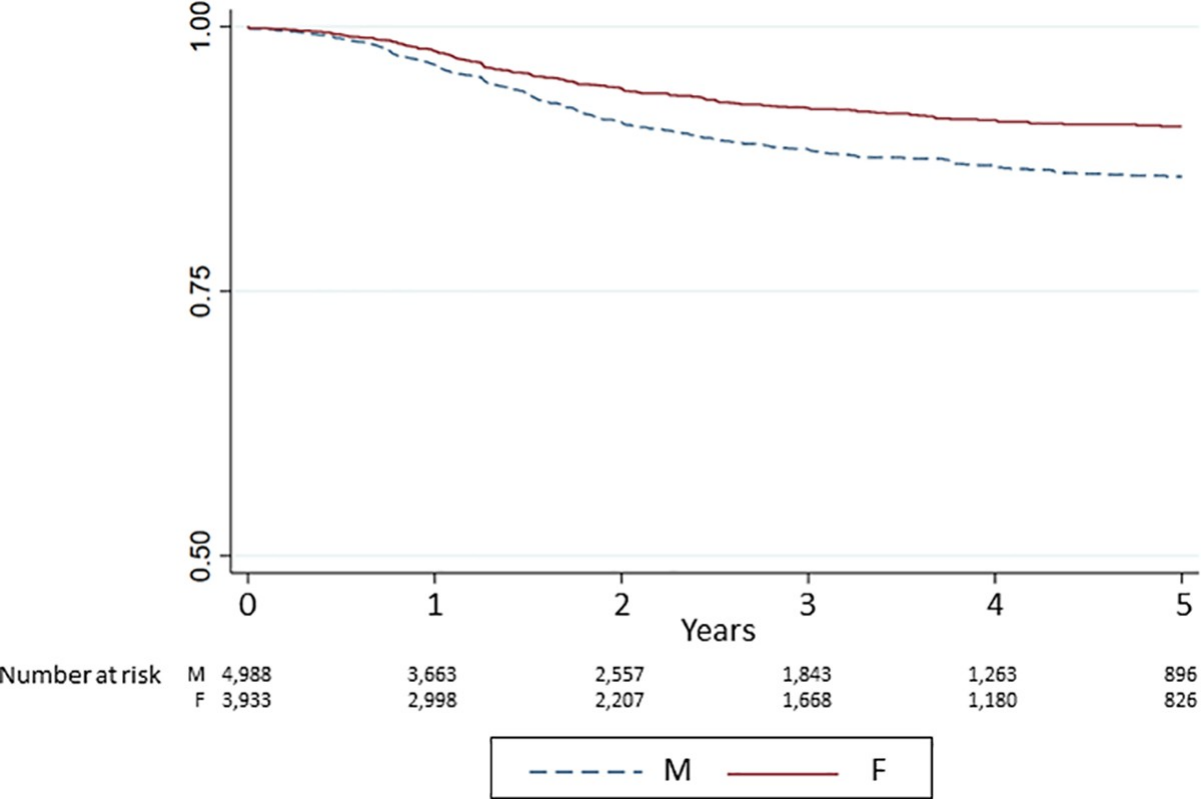
Waitlist access. Cumulative incidence curve of being waitlisted after entering dialysis treatment.

For the transition from *dialysis to waitlist* a strong sex difference emerged (Table 2, Fig 2). Again correction for age reduced the HR from 0.65 (95%CI 0.56–0.76) to 0.84 (0.72–0.98) highlighting the importance of age for this transition step. The consideration of comorbidities did not result in a change of the HR for females compared to males (HR/95%-CI: 0.82/0.70–0.96), but showed that the presence of diabetes resulted in a reduced likelihood of being placed on the waitlist (HR/95%-CI: 0.72/0.6–0.87 for being diabetic vs. non-diabetic). In the final adjusted model the likelihood of women having access to the waitlist was 18% lower than of men (HR/95%-CI: 0.82/0.70–0.96).

The incidence of waitlisting separates during the first year on dialysis between men and women and remains stable after about three years.

**Fig 3:**
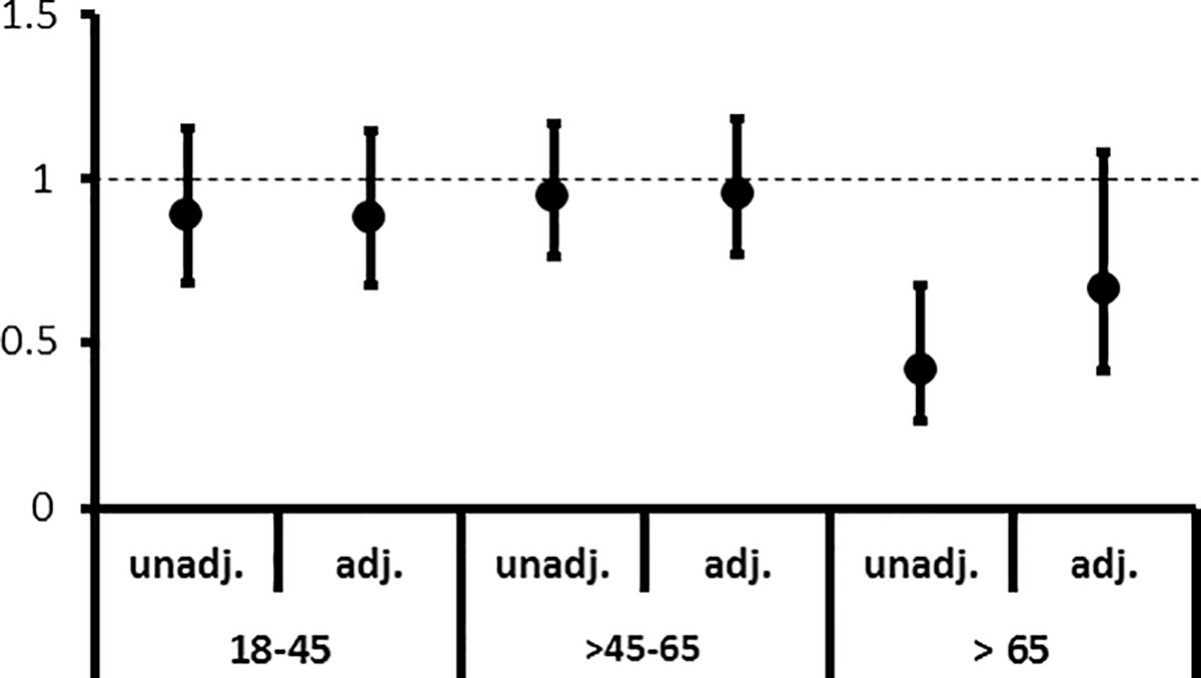
Waitlist access by age group. Hazard ratios (HRs) for the unadjusted (unadj.) model and the model adjusted (adj.) for age and comorbidities.

Because of the importance of age for our model, we stratified the analysis by age groups. In the unadjusted analysis sex difference only emerged in elderly patients over the age of 65 years. No differences between sexes were observed in an adjusted analysis of patients stratified by age (Fig 3). The formal test for interaction was not significant (p = 0.715).

The HRs for being waitlisted after entering dialysis for females compared to males across three different age subgroups 18–45 years, >45–65 years and >65 years are shown. Only for the unadj. analysis of the oldest age subgroup (>65 years) a significant result occurred. After adjustment this difference was no longer visible.

### Waitlist to transplantation

For the final transition from *waitlist to transplantation* no sex differences were found (HR/95%-CI: 0.96/0.81–1.14). This holds true irrespective of the covariates included (Table 2).

### Transplantation outcome

Transplantation outcome was not influenced by sex in the unadjusted analyses (HR/95%-CI: 0.99/0.72–1.34) (Table 2). The introduction of age (HR/95%-CI: 1.2/1.07–1.32) alone as well as the introduction of age together with the comorbidities showed that age (HR/95%-CI: 1.13/1.002–1.27), ischemic heart disease (HR/95%-CI: 1.47/1.02–2.13) and cerebrovascular disease (HR/95%-CI: 1.57/1.07–1.88) were associated with transplant failure (death/graft failure).

**Fig 4:**
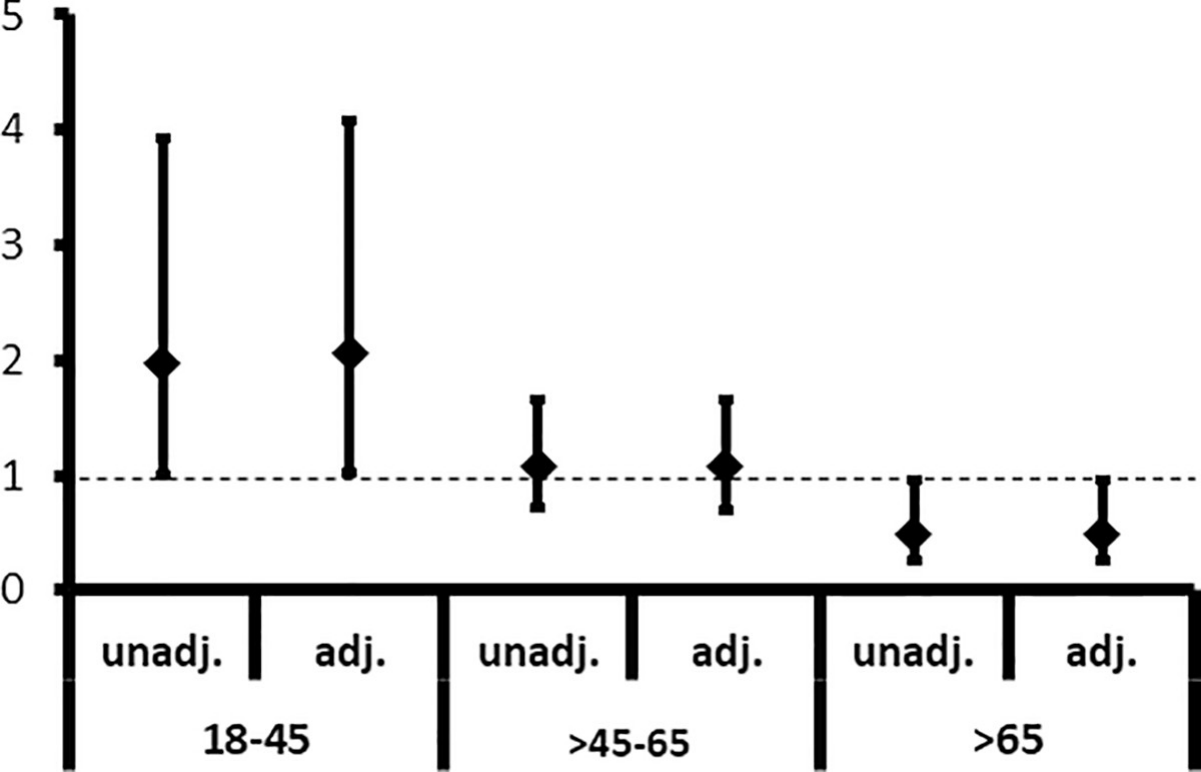
Risk of transplant failure or death.

Stratification by age groups (Table 3, Fig 4), revealed that female patients aged 45 and younger had a more than doubled likelihood (HR/95%-CI: 2.08/1.04–4.14) to experience transplant failure when compared to men. In patients aged between 45 and 65 years there was no difference with regard to transplant outcome (HR/95%-CI: 1.07/0.69–1.65). Female patients older than 65 years experienced transplant failure less likely than men; graft survival in this age group was 53% better in women (HR/95%-CI: 0.47/0.24–0.93). The interaction between sex and age was statistically significant (p = 0.005).

**Table 3:** Hazard ratios for the combined endpoint transplant failure or death.

Age group	18–45 years			>45–65 years			>65 years		
	m: 106 → 15			m: 196 → 52			m: 93 → 41		
	f: 65 → 18			f: 132 → 36			f: 45 → 11		
	Model 1	Model 2	Model 3	Model 1	Model 2	Model 3	Model 1	Model 2	Model 3
**Sex (female vs. male)**	1.98*	2.05*	2.08*	1.09	1.08	1.07	0.49*	0.49*	0.47*
95% CI	1.00–3.94	1.03–4.08	1.04–4.14	0.71–1.67	0.70–1.65	0.69–1.65	0.25–0.96	0.25–0.96	0.24–0.93
**Age (10 years)**		1.25	1.25		1.10	1.02		1.06	1.20
95% CI		0.72–1.82	0.73–1.82		0.72–1.50	0.63–1.42		0.26–1.93	0.37–1.17
**Diabetes**			0.62			0.72			1.40
95% CI			0.14–2.75			0.42–1.25			0.77–2.56
**IHD**			0.41			1.85*			1.47
95% CI			0.05–3.61			1.10–3.13			0.82–2.63
**CVD**			1.95			1.72			1.28
95% CI			0.21–18.4			0.91–3.27			0.67–2.45

Hazard ratios and 95% confidence intervals from regression analyses using Cox Proportional Hazards model are displayed. Model 1 only includes sex, model 2 includes sex and age and model 3 includes sex, age and the comorbidities diabetes, IHD and CVD. Significant results are marked

* p<0.05. IHD, ischemic heart disease; CVD, cerebrovascular diseaseHazard ratios for the unadjusted (unadj.) model and adjusted (adj.) for age and comorbidities.

The HRs for the combined endpoint of reentering dialysis (transplant failure) or death for females compared to males across three different age subgroups 18–45 years, >45–65 years and >65 years are shown. In the younger age group (18–45 years) women had an about 2 fold increased risk (HR/95%-CI: 1.98/1.0–3.94) of transplant failure or death, whereas in the oldest age group (>65 years) their risk was half the risk of man (HR/95%-CI: 0.49/0.25–0.96). Adjustment for age and comorbidities did not change the HRs.

## Discussion/Conclusion

Our analysis on sex disparities regarding renal transplantation takes a population-based approach and covers all transition steps a patient requiring RRT will undergo. This is the first analysis addressing a German population. We show that female patients with CKD are less likely to proceed to dialysis. Similarly, women on dialysis are less likely to be waitlisted for renal transplantation. While the first observation may be advantageous for women, the latter is not. As there are no restrictions in access to RRT in Germany, it has been surprising to find the disparity with regard to waitlisting aggravated in older women. This selection against older women, however, may be the cause for the better outcome after transplantation seen for the same age group. Also unexpected and somewhat more difficult to explain is the worse outcome in younger women.

Our finding that the risk for starting dialysis is lower in women with CKD reflects at least in part a slower progression rate in women. It has been shown that CKD progression in various renal diseases is faster in men than in women [33, 34]. That has been confirmed in a very recent study from several European countries [35]. This sex difference has been attributed to different factors like differences in glomerular hemodynamics, in kidney size and mass as well as nephron number, in RAAS activity, and the effect of female sex hormones [11]. In addition to biological facts, there is some evidence that maintenance dialysis might be started at a lower GFR in women than in men due to a less pronounced increase in serum creatinine levels [34, 36]. The latter could not be evaluated by our study as no laboratory values are available in our data set.

Access to the transplant waitlist is markedly reduced in female patients even after adjustment for age and important comorbidities. Once waitlisted, there is no difference in transplantation access. This result resembles two of the four studies that investigated similar steps: one analyzed the Scottish dialysis cohort [18], the other came from the US and had corrected for the presence of panel reactive antibodies [17]. Comparable data showing the disparity most pronounced in women aged above 65 also came from the US [3]. Whereas in a US study authors corrected for panel reactive antibodies [17], we did not see a difference in access to transplantation after waitlisting between women and men without such a correction. This suggests that the presumed higher immunization rate in women does not seem to influence access to transplantation. We can only speculate that this might be due to improved allocation algorithms allowing for the exclusion of potential harmful donor antigens. In Germany organs are exchanged within the Eurotransplant population assuring a large donor pool that allows timely organ allocation even in sensitized patients. A specific program, the Eurotransplant “Acceptable Mismatch Program”, addresses the need in highly sensitized patients with PRA levels over 80% [37]. Patients allocated through this program do not have longer waiting time when compared to patients without sensitization [38]. We feel that the confirmatory results of our analysis are very important for several reasons. While data from single transplant centers or transplant / dialysis registries are subject to potential selection biases, data from an insurance population are not only considered to be complete, but also collected in a standardized fashion thereby overcoming these problems. In addition, our cohort had by definition full coverage of all health care costs excluding purely economic factors as potential reason for the disparities. Interestingly, two studies from the UK [4, 5] also assumed that the lack of sex disparities was a result of full health care coverage. Given the importance of age demonstrated in our data, another explanation of their results could be the exclusion of patients aged over 65 years [5] or the very low number of transplantations in this patient group [4]. This is also a potential explanation for the difference between the UK and US analyses [1–3], as the US data like our data included a considerable amount of elderly patients.

There is extensive literature on the importance of socio-economic factors besides purely financial reasons with regard to waitlist access and access to living donation. These factors are often differentially pronounced in both sexes and may result in gender differences [39, 40]. Several studies have shown that recipients’ socioeconomic status [41, 42], marital status, educational attainment [43] and related factors influence access to deceased as well as living donor transplantation. Importantly, any of these factors must be considered to be part of the causal pathway between sex and waitlisting and could therefore not have been part of our model building process.

Existing comorbidities seem to be weighted differentially between the sexes. Comorbidities play an important role in accessing the transplantation waitlist. Especially diabetes is associated with a 28% lower chance of being waitlisted and transplanted. Importantly, in the dialysis subpopulation diabetes is more prevalent in women than in men, whereas this ratio is reversed for the waitlisted patients. This is pointing towards a disparity in the selection process for the waitlist, i.e. a diabetic woman is more likely to be excluded from waitlisting than a diabetic man. We find a similar pattern for ischemic heart disease. Other studies have shown disparities with regard to body mass index (BMI): while in men a BMI between 25 to 35 kg/m^2^ facilitated transplantation access, women with a BMI higher than 25 kg/m^2^ had a reduced access to transplantation [44]. While obvious differences in the evaluation and weighting of comorbidities exist in our population, we can only speculate whether this occurs on the physicians’ or the patients’ side. It has been described that in patients on maintenance hemodialysis female sex and older age are associated with more transplantation-associated concerns [45] and that physicians misclassify female and elderly dialysis patients more often falsely as frail [46].

When investigating transplant outcome, we observe an interesting effect modification by age. While in younger adults under 45 years of age, transplant outcome is worse in females than in males, it is identical for adults aged 45 to 65 years and superior for elderly women when compared to their male counterparts. This observation has some similarities to a recent study analyzing the Scientific Registry of Transplant Recipients database [27]. The authors showed that female recipients between 15–24 years transplanted with a kidney from a female donor were more likely to experience graft failure when compared to their male counterparts, whereas for females over the age of 45 years graft failure was less likely than in men. In female recipients of a male donor organ the risk of graft failure was always higher than in male recipients. Unfortunately, the effect of donor sex cannot be examined in our data set, but we believe that the better transplant outcome in the elderly is due to the more stringent selection of these women as seen in our analysis of transplantation access. Potential reasons explaining the inferior transplant outcome in younger women must be considered, such as immune-stimulatory effects of sex hormones [47], the so-called HY-effect in case of a male donor organ [27], a higher incidence of PRA [48] and the different spectrum of underlying diseases, e.g. the higher incidence of systemic lupus erythematodes in women [49].

Our study uses data from a state-wide statutory health-insurance that permitted to analyse sex disparities throughout a patient’s medical career. It is important to show that analyses of such important research questions are possible in this type of data, especially because of its standardized collection, completeness and representation of the population at large. Limitations of our study are the lack of laboratory values and donor specific data. Information which is not relevant for billing is not systematically captured. In addition, there are limited baseline variables for comparison between both sexes, which may have added confounding from unmeasured variables in some of our analyses. Certain nuances of CKD progression and transplantation access are therefore difficult to tease out in this kind of health data research. Furthermore, no subjective data on attitude of the patients towards transplantation, i.e. rejection of such an intervention due to religious or other concerns, is available in this study. In addition, parameters not required for billing of medical services are not part of our data set (e.g. body size, late referral, severity of comorbidities or the underlying renal disease that caused ESRD). However, the key information to define time on dialysis or on waiting list as well as the time-point of transplantation is complete and accurate, because of its direct link to physicians’ or hospitals’ reimbursement. Notably, our analyses of transplant outcome had a low power for detecting changes in HR causing some likelihood that established prognostic factors (e.g. recipients’ diabetes) might not show up as significant. However, the analysis indeed showed the importance of sex, which was the main focus of our study. Finally, we can only detect start of dialysis or renal transplantation, but not the occurrence of ESRD without starting dialysis or being transplanted.

It seems desirable to compare our data with data from other European countries. Register based data on the prevalence of renal disease, dialysis and transplantation are provided by the ERA-EDTA registry, representing data from 52 national and regional European registries [28]; the registry does not contain data from Germany. With regard to sex distribution the ERA-EDTA registry data shows that women make up 40% of the RRT population [50], which is very close to the 44% observed in the prevalent dialysis population in our data set. In Europe, 47% of males and females are transplanted [51]. Unfortunately, from our data we cannot match this prevalence data as we only captured incident transplantations by using the code for the transplantation procedure. As the length of observation determines transplantation rate, it is also impossible to compare the transplantation incidence in our data with the published data on European transplantation rates. Graft survival after a first renal transplant in Europe is close to 90% after 2 years and about 80% after 5 years. As our analyses were focused on the comparison of both sexes and not meant to allow for comparisons of outcome of renal transplantation across Europe, we do provide respective data as part of our analyses. We can, however, estimate from our data that death censored graft survival for any renal transplant (without a restriction to first transplants) was 75% after a mean observation period of 5 years.

In conclusion, we detect sex disparities in access to waitlisting for renal transplantation and with regard to transplant outcome in a population covered by a health care system that allows for equal access to medical treatment irrespective of patient income. The fact that disparities occur at different phases of a patient’s medical path highlights the need to further explore the underlying causes for the described differences and at the same time increase awareness in patients as well as physicians that sex disparities still exist.

## Supporting information

S1 Table Power calculation. (DOCX)

S2 Table Proportional hazards regression models for the transition CKD to dialysis including only patients coded as CKD stage 4 and 5. (DOCX)
