# Supplementary material for: Sex disparities in dialysis initiation, access to waitlist, transplantation and transplant outcome in German patients with renal disease—A population based analysis
Source: PLoS One. 2020 Nov 12;15(11):e0241556. doi: 10.1371/journal.pone.0241556 (PMC7660568; doi:10.1371/journal.pone.0241556)
Supplement: S1 Table — (DOCX) [file pone.0241556.s001.docx]

**S1 Table. Power calculation.**

|  | CKD→ dialysis | Dialysis→ waitlist | Waitlist→ transplant | Transplant→ failure | Transplant→ failure  age 18-45 | Transplant→ failure  age >45­-65 | Transplant→ failure  age >65 |
| --- | --- | --- | --- | --- | --- | --- | --- |
| N | 70,793 | 8,921 | 1,197 | 637 | 171 | 328 | 138 |
| Actual HR female vs male | .82 | .84 | .95 | .99 | 2.05 | 1.08 | .49 |
| Power (%) with actual HR | 100 | 62 | 8 | 23 | 51 | 5 | 67 |
| Power (%) with HR 0.9 or 1.1 | 98 | 28 | 21 | 10 | 5 | 7 | 5 |
| Power (%) with HR 0.8 or 1.2 | 100 | 83 | 70 | 30 | 9 | 18 | 11 |

Power of detecting the actual HR (hazard ratio), a decrease or increase of hazard by 10 % (HR 0.9 or 1.1) or by 20 % (HR 0.8 or 1.2) for the respective binary variable (CKD/dialysis, dialysis/waitlist, etc.) and one linear covariate without correlation. This power analysis was performed using “powerEpi.default” from the R package “PowerSurvEpi”.
