# Supplementary material for: Sex disparities in dialysis initiation, access to waitlist, transplantation and transplant outcome in German patients with renal disease—A population based analysis
Source: PLoS One. 2020 Nov 12;15(11):e0241556. doi: 10.1371/journal.pone.0241556 (PMC7660568; doi:10.1371/journal.pone.0241556)
Supplement: S2 Table — (DOCX) [file pone.0241556.s002.docx]

**S2 Table. Proportional hazards regression models for the transition CKD to dialysis including only patients coded as CKD stage 4 and 5.**

|  | **CKD (stage 4 and 5) -> Dialysis** | | |
| --- | --- | --- | --- |
| Case numbers | **21,856 -> 5,284** | | |
|  | **Model 1** | **Model 2** | **Model 3** |
| **Sex** | 0.75** | 0.82** | 0.85** |
| 95% CI | 0.71-0.79 | 0.77-0.86 | 0.80-0.90 |
| **Age (10yrs)** |  | 0.81** | 0.73** |
| 95% CI |  | 0.79-0.83 | 0.71-0.75 |
| **Diabetes** |  |  | 1.56*** |
| 95% CI |  |  | 1.47-1.65 |
| **IHD** |  |  | 1.40*** |
| 95% CI |  |  | 1.32-1.48 |
| **CVD** |  |  | 1.28*** |
| 95% CI |  |  | 1.20-1.36 |

Hazard ratios and 95% confidence intervals from regression analyses using Cox Proportional Hazards model are displayed. Model 1 only includes sex, model 2 includes sex and age and model 3 includes sex, age and the comorbidities diabetes, IHD and CVD. Significant results are marked: * p<0.05 and ** p<0.001. IHD, ischemic heart disease; CVD, cerebrovascular disease; Tx, transplantation.
